# Prospects and limitations of genomic offset in conservation management

**DOI:** 10.1111/eva.13205

**Published:** 2021-03-18

**Authors:** Christian Rellstab, Benjamin Dauphin, Moises Exposito‐Alonso

**Affiliations:** ^1^ Swiss Federal Research Institute WSL Birmensdorf Switzerland; ^2^ Department of Plant Biology Carnegie Institution for Science Stanford CA USA; ^3^ Department of Biology Stanford University Stanford CA USA

**Keywords:** assisted migration, conservation management, genomic offset, genomic vulnerability, local adaptation, predictive genomics

## Abstract

In nature conservation, there is keen interest in predicting how populations will respond to environmental changes such as climate change. These predictions can help determine whether a population can be self‐sustaining under future alterations of its habitat or whether it may require human intervention such as protection, restoration, or assisted migration. An increasingly popular approach in this respect is the concept of genomic offset, which combines genomic and environmental data from different time points and/or locations to assess the degree of possible maladaptation to new environmental conditions. Here, we argue that the concept of genomic offset holds great potential, but an exploration of its risks and limitations is needed to use it for recommendations in conservation or assisted migration. After briefly describing the concept, we list important issues to consider (e.g., statistical frameworks, population genetic structure, migration, independent evidence) when using genomic offset or developing these methods further. We conclude that genomic offset is an area of development that still lacks some important features and should be used in combination with other approaches to inform conservation measures.

## 1 PREDICTING THE RESPONSE OF SPECIES AND POPULATIONS TO CHANGING ENVIRONMENTS

In a world of anthropogenically induced climate and land use changes, species and populations are often under high pressures to adapt to the new environmental conditions. If these changes are persistent, only migration and adaptation can help a species avoid local extinction. In many fields like nature conservation, forestry, fishery, agriculture, but also generally in science of applied ecology and evolution, there is a keen interest in predicting how populations will respond to environmental changes (Foden et al., 2019). For example, such predictions could be used to estimate whether a population can self‐sustain under future alterations of its native habitat, whether it has to disperse or migrate to track its ecological niche, or whether it may require human intervention such as protection, restoration, or assisted gene flow or assisted migration (see Glossary in Box 1). If populations cannot keep up with the pace of change, predictions can be used in restoration plans that essentially involve the transplant or relocation of genotypes potentially adapted to the target environment. Such spatial and/or temporal predictions may thus ensure the survival and persistence of populations with sufficient local fitness (Aitken & Bemmels, 2016; Aitken & Whitlock, 2013).

BOX 1 **Glossary**

- *Assisted* *gene* *flow*—The intentional movement of gametes or individuals between populations *within* the species’ range to mitigate local maladaptation.
- *Assisted* *migration*—The intentional movement of gametes or individuals between populations *beyond* the species’ range to mitigate local maladaptation.
- *Common* *garden* *experiment*—Also called provenance trial. Planting or moving organisms from different provenances (populations) into a common experimental environment to measure fitness‐related phenotypic traits. Observed differences in traits have a genetic basis.
- *Ecological* *niche* *modeling—*Also called species distribution modeling (but see, Peterson & Soberon, 2012). Using statistical relationships between species occurrence data and environmental data, ecological niche modeling can be used to assess habitat suitability, to identify environmental factors driving the current species distribution, or to predict the species’ potential distribution, for example in unsampled regions or under future environmental conditions.
- *Genetics* *versus* *genomics—*Genetics studies a limited number of loci (e.g., microsatellites, SNPs, genes) that are often well described. However, the term is also used in a general way, for example when talking about genetic vs. environmental effects on a trait. In contrast, genomics investigates patterns at the genome‐wide scale, or at least at a reduced representation of it. This makes it possible to disentangle neutral and adaptive genomic variation by genome scans for selection such as outlier analysis or genotype–environment associations.
- *Genotype–environment* *association* *analysis*—Also called environmental association analysis. The main analytical approach in landscape genomics (see below) that enables the identification of genes and environmental factors presumably involved in environmental adaptation. It statistically correlates environmental factors describing habitats and allele frequencies of populations/individuals, while ideally accounting for the confounding population genetic structure. Loci with a significant correlation are putatively involved in adaptation to the tested environmental variable. This approach assumes that populations are adapted to their local environment.
- *Genotype–phenotype* *association* *analysis*—Also called genome‐wide association study (GWAS). Correlating allele frequencies and trait values of numerous individuals in a common garden (see above) to pinpoint genomic regions possibly underlying the investigated phenotypic traits. Such analyses stringently control for relatedness and population genetic structure.
- *Genomic* *offset*—The distance between the current and required genomic composition in a set of putatively adaptive loci under a future/changed environment. The latter can be understood in a spatial or temporal perspective. For examples of genomic offset methods, see Box 2.
- *Landscape* *genomics—*Landscape genomics combines genomic and environmental data to detect and describe signatures of selection in the genome, which is also called adaptive genomic variation. Its name originates from the field of landscape genetics, where the effect of landscape features on neutral genetic variation, differentiation, and structure is studied.
- *Space*–*for*–*time* *substitution* *approach*—The use of spatial data to infer temporal dynamics in the absence of temporal data. Spatially separated sites along ecological or environmental gradients serve as proxies for predicting time series.

In the past, assessments of persistence in a different or future habitat have been mainly based on assessing fitness (e.g., survival and fecundity) in common garden experiments (Box 1) or on ecological niche modeling (Box 1) using habitat suitability as proxy (Elith & Leathwick, 2009). Both approaches have limitations. Experiments are resource‐consuming and often impractical for long‐lived or protected species. Ecological niche models require very little data and are thus widely applicable, but they do normally not account for demographic (e.g., different genetic clusters) or evolutionary (e.g., different ecotypes) properties of the species (but see Exposito‐Alonso et al., 2018; Razgour et al., 2019). Therefore, there is a need for incorporating genomic information into predictions (Waldvogel et al., 2020). Genomic data (Box 1) does not only allow to accurately describe (neutral) genetic diversity, differentiation, and population structure, but also enables to track signatures of selection in the genome, which can inform us about the impact of environmental pressures on populations (Savolainen et al., 2013; Stapley et al., 2010), and to incorporate knowledge of local adaptation of populations into predictive models (Razgour et al., 2019).

An increasingly popular predictive approach that accommodates putative evolution and adaptation of populations to the new environment is genomic offset (reviewed in Capblancq et al., 2020). It is also known as genetic offset (Fitzpatrick & Keller, 2015), genomic vulnerability (Bay, Harrigan, Le Underwood, et al., 2018), or risk of nonadaptedness (Rellstab et al., 2016), but here we refer to "genomic offset" to emphasize its genomic perspective. Today, there are a few dozens of studies, mainly focusing on forest trees (Borrell et al., 2019; Ingvarsson & Bernhardsson, 2020; Pina‐Martins et al., 2019), but also on animal systems (Bay, Harrigan, Le Underwood, et al., 2018), that have used genomic offset to predict the possible fate of populations with a particular focus on future climate change. Using genomic and environmental data from geo‐referenced individuals, genomic offset normally first establishes a statistical relationship between an environmental factor and allele frequencies of populations or individuals (by genotype‐environment association analysis, reviewed in Rellstab et al., 2015) and then spatially and/or temporally predicts the genetic composition needed for the (modeled) new or future environment (habitat) of the populations. In doing so, genomic offset can assess the degree of possible maladaptation of these populations to their new environmental conditions. Similarly, it can also quantify the potential nonadaptedness of a population to its current environment (Borrell et al., 2019).

Such an intuitively simple and apparently powerful approach has readily been suggested to be adopted for conservation management (Capblancq et al., 2020). Here, we highlight the prospects and various limitations of genomic offset, the latter being especially important when used for recommendations in conservation, assisted migration, or other applied activities. However, genomic offset is also a valuable tool for research that is not directly linked to conservation, such as exploring the genetic architecture of adaptive traits. Our perspective aims to contribute to the debate on current issues in the field (Fitzpatrick et al., 2018; Rellstab, 2021).

## 2 THE CONCEPT OF GENOMIC OFFSET

Based on a statistical genotype–environment relationship (Box 1) derived from past or contemporary data of locally adapted populations (Rellstab et al., 2015), one assesses the genomic composition needed for the measured new and/or modeled future environment (Box 2). Importantly, only loci and genes that are putatively involved in adaptation to the studied environmental drivers are used for such calculations. The genetic distance between the present and the predicted theoretically required genomic composition represents the genomic offset. If this offset is small, populations are expected to have a low risk of being maladapted to their future habitats. If the offset is large, populations are far away from their future, theoretically required genomic composition and may be at high risk of maladaptation. Genomic offset is generally restricted to environmental and genotypic data, thus not including any phenotypic traits, but extended approaches have been recently developed (Box 2). Genomic offset can be assessed in a spatial and/or temporal manner. Often, the genotype–environment relationship is first extrapolated in space and subsequently projected for future environmental conditions. This allows the assessment of genomic offset also for habitats that were not sampled when establishing the genotype–environment relationship.

BOX 2 **Graphical illustrations and statistical frameworks of the main genomic offset approaches**

The terms “model,” “modeling,” or “predicting” are commonly used to refer to the usage of a mathematical equation to characterize the relationship between different variables. In our case, these variables are genotype (i.e., allele frequencies) and environment (i.e., a variable describing the habitat of the genotype). These models can then be applied to extrapolate to unsampled environments. Such methods were inspired by phenotype‐based equivalents where phenotypes of different provenances in common gardens are associated with their environment of origin with a linear regression, which is then used to predict the future phenotypic responses (risk of maladaptation, Box 2a).

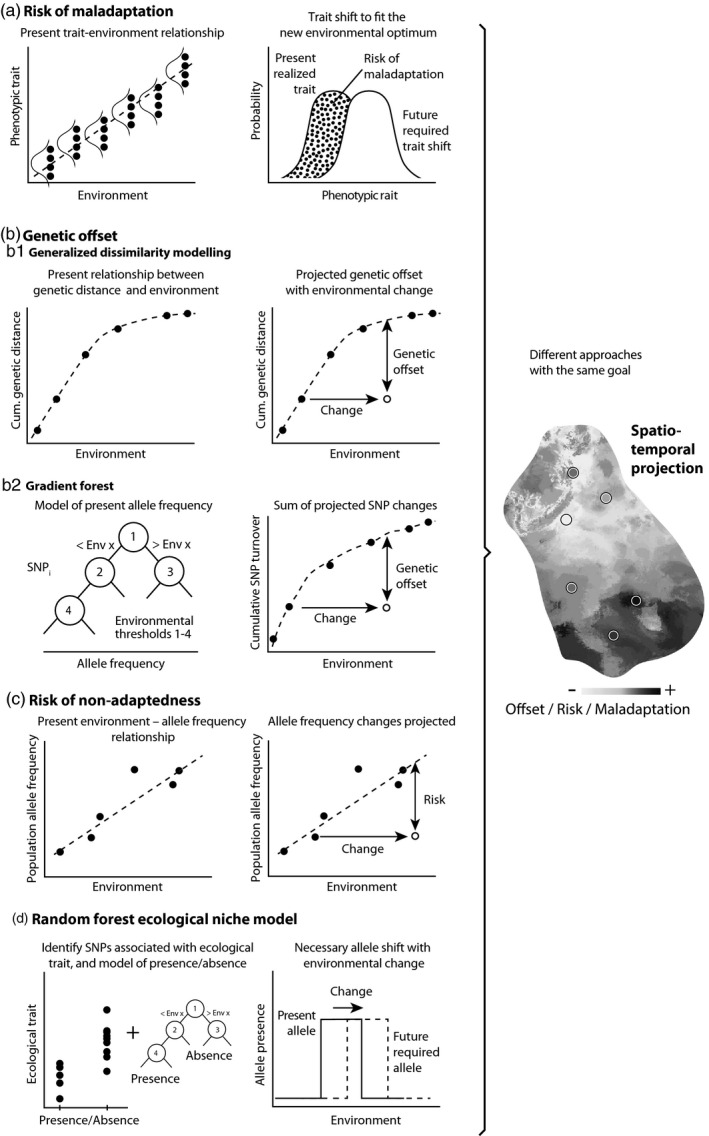

Below, we describe the two fundamental steps—the genotype–environment association and the spatial/temporal extrapolation—used in different genomic offset approaches and the statistical models behind them.

**Prestep: Genotype–environment associations**

The simplest model to describe the relationship between genetic and environmental characteristics of populations is the linear regression:

(1) $$Y_{i}=\alpha +\beta \cdot E+\varepsilon$$

where we want to identify *Y_i_*, the genetic composition (allele frequency) of a focal population *i*, by knowing *E*, its environmental characteristics. Using populations for which we know the genetic composition and environmental conditions, we can estimate the best values of the intercept and slope coefficient of the linear regression and then use those fitted values to predict *Y* for any *E*. This approach should account for neutral population genetic structure, which is often done using a mixed model that is an extension of the regression model:

(2) $$Y_{i}=\alpha +\beta \cdot E+u+\varepsilon$$

where *u* (random factor) is the genetic similarity between populations at neutral loci. These associations serve researchers to narrow down a genomic dataset to a subset of candidate genetic variants that can be used for genomic offset predictions.

**Main step: Genomic offset fit and prediction**

**Gradient‐based (Box 2b)**

The original study of genetic offset (Fitzpatrick & Keller, 2015) proposed two approaches. In the Generalized Dissimilarity Modeling, the predicted variable is a composite of allele frequency differences (*d_ij_*, which is the average *F*_ST_) between two populations at the loci of interest. These loci derive from known candidate genes or identified using the prestep of genotype–phenotype associations described above. The predictive variables are environmental distances that are modeled as follows:

(3) $$‐\ln\left(1‐d_{\mathrm{ji}}\right)=a_{0}+\sum_{p=1}^{n}\left|f_{p}\left(x_{\mathrm{pi}}\right)‐f_{p}\left(x_{\mathrm{pj}}\right)\right|$$

where *i* and *j* are sites, and the distance *d_ij_* is modeled with an intercept and a spline function *f* of the *n* different environmental values *x*.

The second approach, Gradient Forest, models the change of allele frequency at each locus along an environmental gradient. Then, the predictions are summarized across all variants. Gradient Forest is an extension of Random Forest, an ensemble of decision trees that split the dataset into two population groups each time with the same frequencies by one environmental variable at each bifurcation. At each bifurcation, the algorithm chooses the value of an environmental variable that increases the variance reduction of the variable before splitting the data into two groups (*V*_0_) and after the sum of the variances of the group over a threshold and before the threshold:

(4) $$I=V_{0}‐\left(V_{T}+V_{F}\right)$$

**RONA—Risk of nonadaptedness (Box 2c)**

RONA (Rellstab et al., 2016) uses a linear regression in previously identified, putatively adaptive loci as shown in formula (1) without direct correction for population structure to estimate the expected allele frequency required under the new environmental conditions (*E*_expected_). However, population structure can be accounted for by using transformed or residual allele frequencies after accounting for structure. RONA is then calculated as the average difference (across *n* loci) of expected and observed (AF_observed_) allele frequencies in a population:

(5) $$\text{RONA}=\frac{1}{n}\sum_{i=1}^{n}|(\alpha +\beta *E_{\text{expected}}{\text{) ‐ AF}}_{\text{observed}}|$$

Pina‐Martins et al. (2019) expanded RONA by using a weighted average by the *R* ^2^ of the regression.

**Random forest ecological niche models (Box 2d)**

Exposito‐Alonso et al. (2018) used a discrete approach, using environmental niche models (typically used to predict species presence and absence), to model allele presence and absence. This was done iteratively for ca. 100 bi‐allelic sites, and the output maps were summed over (as in Gradient Forest). A popular statistical procedure to fit an environmental niche model is the Random Forest, a machine learning approach (but others such as the Maximum Entropy algorithm are common too). In brief, the algorithm proposes a threshold for a predictor variable to group observations of geographic presence and absence of the species. In the simplest case of a decision tree with a single bifurcation (a single climate threshold), one can evaluate the predictive ability of grouping presence and absence points using the Gini impurity index:

(6) $$1‐\frac{1}{2}\sum_{i\in \left\{p,a\right\}}p_{i}^{2}‐\frac{1}{2}\sum_{i\in \left\{p,a\right\}}q_{i}^{2}$$

where *p_a_* and *p_p_* represent the proportion of real presences and absences in the alleged “presence” group, and *q_a_* and *q_p_* the same for the alleged “absence” group; the index is zero with perfect partition. The algorithm will find the threshold that reduces the Gini index and continues bifurcating with thresholds of other climate variables to improve the classification of observations.

Different research groups have independently developed similar “genomic offset” approaches. Conceptually, genomic offset is analogous to the so‐called "relative risk of maladaptation" (Box 2a), which was developed using phenotypic data (not genomic data) for seed transfers in forest trees (Campbell, 1986). The relative risk of maladaptation was made popular by the study of St. Clair and Howe (2007), who measured various phenotypic traits in a common garden experiment using Douglas‐fir (*Pseudotsuga* *menziesii*), and compared the fitted normal distribution of the present trait values to the one ideal at the target locations based on phenotype–environment associations. In essence, this empirical approach follows the theoretical stabilizing selection model of a moving trait optimum analytically studied by Lynch and Lande (1993). The area of the original trait distribution, which is not overlapping with the predicted distribution, represents the relative risk of maladaptation at the target location (Box 2a). By incorporating the temporal dimension, this concept was subsequently used to predict the risk of maladaptation to future climatic conditions (Frank et al., 2017).

Fitzpatrick and Keller (2015) were the first to present a genomic offset approach (calling it "genetic offset"). Using tools borrowed from community ecology—generalized dissimilarity modeling and gradient forests (Box 2b)—they established a statistical relationship between environment and allele frequencies in single‐nucleotide polymorphisms (SNPs) included in the circadian clock gene *GIGANTEA*‐*5* of balsam poplar (*Populus* *balsamifera*). They first extrapolated this relationship to the present range‐wide space and subsequently to a projected future climate. In doing so, they indicated geographic regions where the risk of maladaptation might be low or high based solely on genomic and climate information. Right away, this technically intuitive analysis of genomic data with powerful geographic visualization and interpretation was rapidly adopted, especially the gradient forest model, most likely due to its nonlinear nature with a machine learning‐based framework. For example, Bay, Harrigan, Le Underwood, et al. (2018) predicted climate‐driven genetic offset (which they called "genomic vulnerability") in a migratory bird species (yellow warbler, *Setophaga* *petechia*) and compared it to past population declines. This article started a critical and constructive discussion on how to use and interpret the concept with its underlying pitfalls and benefits (Bay, Harrigan, Buermann, et al., 2018; Fitzpatrick et al., 2018), which builds also the basis and motivation of this current perspective paper.

Rellstab et al. (2016) used a similar approach called "risk of nonadaptedness" (RONA) to predict the theoretically required allele frequency shifts of various populations of different white oak species (*Quercus* spp.) for future climate change (Box 2c). These authors did not model RONA in a spatially explicit manner (although this would be possible), but instead predicted the offset for every sampled population. Unlike the genetic offset of Fitzpatrick and Keller (2015) that uses a composite genetic distance metric, RONA directly predicts the required allele frequency shift in a population to be adapted to the future conditions, making it possible to compare these presumably required allele frequency shifts to the past realized, theoretically expected (Dauphin et al., 2021), or even future realized values, for example, if populations are re‐sequenced in the future. RONA has been regularly used (Jordan et al., 2017), was recommended as a tool in assisted gene flow (Borrell et al., 2019), and is available as a Python implementation (Pina‐Martins et al., 2019).

Recently, a similar approach to the gradient forest framework of Fitzpatrick and Keller (2015) has been implemented using random forests of allele presences and absences across a species’ distribution range (Exposito‐Alonso et al., 2018). In this study, hundreds of thale cress (*Arabidopsis* *thaliana*) plants were grown in a common garden experiment under a drought stress treatment, and alleles involved in survival were identified using genotype–phenotype associations (Box 1). The distribution of about 100 alleles was modeled with random forest akin to species distribution models and was spatially predicted in present and future climates. Similar to the original genomic offset approaches, the comparison between these predictions was interpreted as the expected allelic composition change in the near future across Europe (Box 2d). While costly, the use of common gardens allows both to conduct genomic offset predictions with bona fide adaptive alleles and to ultimately validate genomic offset predictions through space (Exposito‐Alonso et al., 2019).

## 3 IMPORTANT ISSUES TO CONSIDER WHEN USING GENOMIC OFFSET APPROACHES

Genomic offset approaches are in their infancy of development and application. Consequently, they have yet to be tested and validated experimentally in scenarios simulating real conservation applications. Below, we enumerate a number of key assumptions, elements that will require further development, or methodological pitfalls that need to be considered before the application of genomic offset methods for conservation management.

### 3.1 Space‐for‐time substitution approaches

Genomic offset represents a "space‐for‐time substitution approach" (Box 1). However, we have to be aware that what we observe across space might not hold true over time. This is because spatial genetic patterns are the outcome of local natural selection as well as neutral demographic processes such as migration, population expansion/contraction, or admixture. Ideally, researchers would validate the space‐for‐time substitution assumption using temporal series of data such as "evolve and re‐sequence" experiments (Long et al., 2015), biological archives (e.g., zooplankton resting eggs in lake sediments, Brede et al., 2009; Rellstab et al., 2011), historical samples (e.g., fish otoliths, Therkildsen et al., 2019), herbarium specimens (Lang et al., 2019), or different age cohorts of long‐lived species (e.g., trees, Dauphin et al., 2021; Elleouet & Aitken, 2019). Moreover, the space‐for‐time substitution approach (and notably genome scans for selection in general) assumes that populations are currently adapted to their local habitat but it has been shown that this might not always be the case (see below and Browne et al., 2019; Leimu & Fischer, 2008).

### 3.2 Spatial and temporal extrapolation

The genomic offset approach assumes that environment–allele frequency relationship is at local adaptation equilibrium and holds beyond the present climatic values and genetic background. However, we do not know if what we observe in a given environmental space holds also true outside of it. Since species will often encounter environments outside their current range in the future, genomic offset predictions into these ranges should be treated with caution and at least be acknowledged in analyses and maps. The same applies to spatial extrapolations in the present, which often come with an extension of the genomic background due to isolation by distance or new genetic lineages. Even at a regional scale—within the same species—the genetic basis of climate adaptation can differ substantially (Rellstab et al., 2017), especially when adaptation is polygenic, that is, there are multiple redundant paths to an optimum (Höllinger et al., 2019, and see below).

### 3.3 Effect of population genetic structure

Only one of the existing genomic offset approaches directly incorporates neutral population genetic structure (Gain & François, 2020). However, we know that neutral genetic structure can confound genotype–environment association analyses and even mimic adaptive patterns (see examples in Rellstab et al., 2015). Fitzpatrick and Keller (2015) used Moran's eigenvector map variables to incorporate spatial autocorrelation into the generalized dissimilarity modeling approach (but not to the gradient forest approach). This assumes that population structure is somehow correlated with geographic position, which might not be the case in species with complex demography and highly heterogeneous habitats. Future studies should therefore directly incorporate or at least evaluate the effect of such a confounding neutral structure. It is clear that the future demography is unknown, which means that we have to assume that current neutral structure remains the same in the future. One possible way to include neutral structure is to use mixed models (with random factors describing structure) for the statistical prediction of the genomic offset (Gain & François, 2020). An alternative is to use transformed allele frequencies or allele frequency residuals after accounting for neutral structure (Gautier, 2015; Günther & Coop, 2013). Another possibility to assess the effect of the confounding structure is to run both random and associated SNPs and compare the outcomes for both types of loci (Exposito‐Alonso et al., 2018).

### 3.4 Migration and gene flow

To go one step further than assessing the required allele frequency changes in situ and solely based on standing genetic variation, genomic offset should account for or incorporate demographic properties such as migration and gene flow. For example, migration distance could be incorporated in analyses. In addition to the "local offset," Gougherty et al. (2021) present an interesting analytical approach ("forward offset") in which they measure the geographic distance of the local, present population to the location where the genomic offset is minimized under future climate conditions. Such an approach relies on additional, sensitive assumptions (e.g., concerning migration distance, or that populations ideally migrate to the location with minimized risk of maladaptation), but might deliver additional evidence on the range and geographic direction of future habitats (Rellstab, 2021). Gene flow, whether beneficial or detrimental, could be accounted for by the geographic position and genetic similarity of proximal populations. The incorporation of migration and gene flow might substantially improve the predictive power of genomic offset assessments. However, this additional step is not always needed, for example in the case of selecting source populations for assisted migration or gene flow, where migration and dispersal are actively managed by humans. In this case, the geographic distance between the source and target locations might be considered in addition to offset values (Borrell et al., 2019), as there could be local adaptation to environmental variables that were not included in analyses.

### 3.5 Validation with empirical and simulated data

Genomic offset analysis should be designed to incorporate time series to describe allele frequency shifts post hoc or observe them in real time. For example, one could sample two different age cohorts (past, today) from several populations, perform genotype–environment association analysis using the past cohorts only and check whether the identified beneficial alleles have actually increased in frequency in the cohorts of today. Similarly, one could predict the offset of the past cohort for conditions of today and check whether the genotypes that had the highest offset values are absent from the present population, or even compare it to the history of census or effective population size. Several study systems (see above) would qualify for such a study, and interesting projects to follow allele frequency shifts have been initiated (see, e.g., http://grene‐net.org/). If this is not possible, simulations could be used to assess the relative power of different genomic offset approaches and to test the effect of demographic properties and sampling designs on the outcomes, as it has been done for other population and landscape genomic methods (De Mita et al., 2013; Forester et al., 2016; Lotterhos & Whitlock, 2015; de Villemereuil et al., 2014). Such studies are completely lacking so far.

### 3.6 Corroboration with independent data

Genomic offset studies should combine their results with other independent, ecological data, if available. For example, Bay, Harrigan, Le Underwood, et al. (2018) found a correlation between genomic offset for future conditions and contemporary population decline in the yellow warbler, indicating that failure to adapt may have already negatively affected populations (but see Bay, Harrigan, Buermann, et al., 2018; Fitzpatrick et al., 2018). Borrell et al. (2019) found that dwarf birch populations (*Betula* *nana*) with high RONA values were particularly small, isolated, and at the margins of the species’ distribution. Such links between independent datasets are very interesting, can compensate for the lack of phenotypic traits, and might strengthen the results obtained. At best, genomic offset approaches are combined with phenotypic data in common garden or reciprocal transplant experiments. Borrell et al. (2019) also analyzed fitness‐related phenotypic traits in a common garden. They found a significant negative relationship between RONA (not calculated for the future climate, but the climate of the common garden) and reproductive output (no. of catkins) of the dwarf birch provenances. Similarly, Fitzpatrick et al. (2021) showed an inverse relationship between genomic offset and performance in two common gardens and that genomic offset better predicted performance than climate distances. A further step would then be to show that certain alleles are beneficial in certain environments (Exposito‐Alonso et al., 2019). However, the missing evidence for fitness relevance of the loci used for many genomic offset calculations is an important point that is not specific to genomic offset studies, but also to many other approaches like genotype–environment association analysis or outlier tests (for selective sweeps) that do not look at phenotypes. We are aware that for most of the study systems, especially for protected or long‐lived species, the goal of combining genomic offset studies with analysis of fitness‐relevant traits is out of reach. The accumulation of experimental evidence in multiple model species can thus be key to assess how accurately genomic offset predictions mirror populations’ fitness or demographic traits.

### 3.7 Choice of the statistical framework

The properties of the study species, available data, and research question may determine which statistical framework to calculate genomic offset may be most suitable (Box 2). For instance, study systems in which adaptation is expected to be highly polygenic (see below) may require genetic offsets that sum over loci genome‐wide (i.e., gradient forest or genetic dissimilarity models), whereas studies using sampling setups with clear, single selection pressures could use single‐loci models (RONA). If genetic structure or climate gradients are sharp, nonlinear genotype–environmental relationships may be more appropriate (gradient forest or random forest), while if gradients are smooth, then linear, regression‐based models may be appropriate (RONA). If phenotypic data from common gardens are available, random forest ecological niche models can be applied. With the ongoing development and increasing use of machine learning methods, which can approximate any relationship, we expect those to become the most commonly used (Vanhove et al., 2021), with the possible cost of over‐fitting the models. In any case, it is important to be aware of the pros and cons of each approach and to understand the uncertainty that comes with it.

### 3.8 Quantification of model uncertainty

Genomic offset studies should quantify, report, and account for the uncertainty and power of the underlying models. In other words, cross‐validation values (e.g., in gradient/random forest methods) or regression statistics (e.g., *R* ^2^ in RONA) should be indicated in order to judge the quality of the offset calculation. Unfortunately, this has been rarely done so far. For example, for the gradient forest model used in Bay, Harrigan, Le Underwood, et al. (2018), no indication of overall model performance is mentioned. If cross‐validation values were low already in the training populations used to build the model, the spatial and temporal extrapolation of it would be highly imprecise. Cross‐validation in machine learning methods such as gradient or random forests should be done carefully, for example based on "leave‐population‐out" instead of "leave‐individual‐out" (i.e., spatial versus random cross‐validation), because the latter can artificially inflate goodness values due to the lack of independence of the training and test datasets (Meyer et al., 2019). If possible, final genomic offset calculations should account for these values, as in Pina‐Martins et al. (2019) who used weighted average (by *R* ^2^) to indicate average RONA values across loci.

### 3.9 Quantification of allele frequency shifts to increase interpretability

Researchers should aim to use population genetic parameters to quantify how populations respond to environmental change. For example, we may want to know the rate of allele frequency shift (i.e., allele frequency changes per time unit) at neutral and adaptive loci in the wild, which is a function of generation time, gene flow, effective population size (*N*_e_), and selection strength in the case of adaptive loci. Allele frequency shifts can be assessed by forward‐in‐time population genetic simulations, but should, if possible, be empirically validated in the studied system. This was precisely done in Dauphin et al. (2021) where the required allele frequency shifts (i.e., RONA) of populations of a long‐lived conifer tree species (Swiss stone pine, *Pinus* *cembra*) for future climate change were substantially higher than historically realized and simulated allele frequency shifts. Other population genetic parameters, such as various genetic distance metrics, often only allow qualitative or relative comparisons between populations or climate scenarios.

### 3.10 Polygenic adaptation

Adaptation in nature is often polygenic, that is, it involves many loci (Csillery et al., 2018; Pritchard & Di Rienzo, 2010). Genomic offset approaches should therefore be explicitly extended to modeling polygenic adaptation to capture complex adaptive responses. Machine learning approaches like the gradient forest approach (Fitzpatrick & Keller, 2015) are ideal for analyzing many loci jointly (Brieuc et al., 2018). Other options are multivariate (Carvalho et al., 2020) or vector‐based methods (Borrell et al., 2019). At the same time, genomic offset studies should increasingly look at the genome‐wide level as in Exposito‐Alonso et al. (2018). Studying genetic variation in one (Fitzpatrick & Keller, 2015) or a group of genes (Rellstab et al., 2016) is certainly interesting and straight‐forward, but neglects the genomic and physiological complexity that species possess.

### 3.11 Maladaptation to current environmental conditions

Genomic offset methods are not only useful in predicting potential maladaptation to new environmental conditions, but also to the local conditions observed today. Particularly small, fragmented, and relict populations may have shifted away from their current optimum for the local environmental conditions due to genetic drift, resulting in currently maladapted populations. Genomic offset methods could therefore be used to measure the genetic distance of specific populations to their currently required genetic composition. This was exactly done in the study of Borrell et al. (2019), who differentiated between c‐RONA (the risk of nonadaptedness to the current conditions) and f‐RONA (the one to the future or new conditions) in dwarf birch. Such an analysis could be performed on a "leave‐population‐out" basis, where the offset of the target population is calculated based on the genotype–environment relationship of the remaining populations. Apart from genetic drift, another reason for being maladapted to the current habitat is the past change in environmental conditions. This is especially true for long‐lived species whose populations were established decades or even centuries ago and whose selection pressure is highest during the early life stages. In such cases, genomic offset studies should, if possible, use historical environmental data to establish the genotype–environment relationship (Dauphin et al., 2021; Rellstab et al., 2016).

## 4 CONCLUSIONS: IS GENOMIC OFFSET READY TO BE APPLIED FOR CONSERVATION MANAGEMENT?

Genomic offset combines genomic and past, present, and future environmental data to efficiently assess possible maladaptation of populations to their future habitat. It could be used to, for example, identify threatened populations, select source populations for assisted migration/gene flow or re‐colonization, and identify suitable habitats under a future and altered environment. Any improvements or complementary approaches to existing methods like ecological niche modeling is a gain. Genomic offset adds the evolutionary dimension to management assessments, something that is urgently needed (Waldvogel et al., 2020).

However, genomic offset is an area of active development undergoing experimental validation. Relatively few studies have yet used this approach, and it is mostly unknown how congruent the results are between analytical strategies (e.g., sampling designs, methods) used for initial genotype–environment associations or approaches chosen for offset calculations. Moreover, some general issues that apply to most population and landscape genomic methods, for example the missing link to fitness‐relevant traits, clearly reduce its value to guide conservation decisions. Therefore, we invoke the precautionary principle when dealing with these predictions and recommend using genomic offset along with other approaches (e.g., experiments, ecological niche modeling, genetic diversity assessments) and datasets (e.g., time series, phenotypic data, population trends) from the conservation biology toolkit and accompanying it with experienced expert and local community knowledge on the focal species and habitat. Genomic offset may be especially convenient in nonmodel, protected, or long‐lived species where experiments are impractical or even impossible. Moreover, where fast decisions are needed, for example in the case of highly threatened species with rapidly declining populations, genomic offset might be one of the only possibilities to inform conservation management. In such scenarios, the trade‐off between the ongoing development of the approach and the urgent need for action has to be carefully discussed. In contrast, in situations where time is not so scarce and experiments are possible, it might be wiser to carefully validate the genomic offset assessments. It is also important that genomic offset assessments do not rely on absolute offset values, but are rather used to compare different (climate) scenarios and populations in their risk of maladaptation. This would also allow to judge the uncertainties of such predictions, which is an important information that researchers should communicate to practitioners. In the meantime, we and other researchers will continue to develop and test genomic offset methods to make them trustworthy and useful tool to make conservation and restoration efforts more targeted, precise, and evolution aware.

## CONFLICT OF INTEREST

The authors declare no conflict of interest.

## Data Availability

This article contains no data.
